# Protein Phosphatase Magnesium Dependent 1A (PPM1A) Plays a Role in the Differentiation and Survival Processes of Nerve Cells

**DOI:** 10.1371/journal.pone.0032438

**Published:** 2012-02-27

**Authors:** Meytal Shohat, Daniella Ben-Meir, Sara Lavi

**Affiliations:** Department of Cell Research and Immunology, Tel Aviv University, Tel Aviv, Israel; Sackler Medical School, Tel Aviv University, Israel

## Abstract

The serine/threonine phosphatase type 2C (PPM1A) has a broad range of substrates, and its role in regulating stress response is well established. We have investigated the involvement of PPM1A in the survival and differentiation processes of PC6-3 cells, a subclone of the PC12 cell line. This cell line can differentiate into neuron like cells upon exposure to nerve growth factor (NGF). Overexpression of PPM1A in naive PC6-3 cells caused cell cycle arrest at the G2/M phase followed by apoptosis. Interestingly, PPM1A overexpression did not affect fully differentiated cells. Using PPM1A overexpressing cells and PPM1A knockdown cells, we show that this phosphatase affects NGF signaling in PC6-3 cells and is engaged in neurite outgrowth. In addition, the ablation of PPM1A interferes with NGF-induced growth arrest during differentiation of PC6-3 cells.

## Introduction

Ser/Thr phosphatases can be divided into two major families, the PPP family (containing the PP1, PP2A and PP2B subfamilies) and the PPM family (that contains the PPM1 subfamily, formerly PP2C). The two groups are distinguished by several differences: PPMs consist of monomeric, Mg^2+^-dependent phosphatases, while PPPs are multi-subunit enzymes [1], [2]. The PPM1 family of phosphatases is insensitive to any known inhibitor. To date, at least 16 distinct PPM1 genes have been found in the human genome, which encode for at least 22 isoforms [3]. Members of the PPM1 family are highly conserved in evolution as evident from the growing list of orthologs reported in both higher and lower eukaryotes [4]. The role of PPM1A (formerly PP2Cα) in regulating stress response pathways is well established. The involvement of PPM1A in negative regulation of various stress-induced pathways via the mitogen-activated protein kinase (MAPK) was shown in budding yeasts, fission yeasts, plants and mammals (reviewed in3). These phosphatases were also reported to participate in various other cellular signaling such as cell cycle, DNA checkpoint, growth related pathways and apoptosis [5], [6], [7], [8], [9], [10], [11], [12]. Our research focuses on PPM1A, the most characterized member of the PPM1 family. We have previously shown that overexpression of PPM1A in HEK293 cells can lead to cell cycle arrest in the G2/M phase and to apoptosis [10],[11].

PPM1A mRNA and protein are highly expressed in different types of cells in the brain. PPM1A pattern of expression is different from those reported for other phosphatases, for example PP2B [13],[14]. However, very few neural substrates of PPM1A have been identified [15].

The PC12 cell line is a model for studying neuronal differentiation, survival and signaling [16]. Upon NGF treatment, PC12 cells differentiate into sympathetic neuron-like cells, characterized by neurite outgrowth and expression of many neuronal specific proteins [17],[18]. This differentiation process is accompanied by rapid proliferation for 2–3 days followed by growth arrest [17],[19],[20]. NGF belongs to the neurotrophin family of growth factors. It binds mainly to the TrkA receptor tyrosine kinase and leads to its activation. Activated TrkA receptor further stimulates various signaling cascades, including the phosphatidylinositol 3 kinase (PI3K) and the RAS-MAP kinase pathways [17],[21],[22]. It has been well established that NGF activates the ERK, JNK and p38 mitogen-activated protein kinases pathways through the activation of RAS [23],[24]. The main second messenger of the PI3K pathway is the serine/threonine kinase AKT [22]. Using inhibitors of PI3K it was demonstrated that AKT activity is necessary for NGF induced survival of PC12 cells. Additional downstream second messengers of PI3K were described. These include p70s6 kinase, certain isoforms of protein kinase C and the Rho family of small GTPases [21],[22].

In this study we investigated the role of PPM1A in the regulation of cell cycle, neuronal differentiation and signaling using the PC6-3 cell line. PC6-3 is a subclone of PC12 cells, which was previously shown to differentiate in response to NGF [19]. These cells stably express tetracycline (Tet) repressor and PPM1A under control of CMV promoter/tetracycline operator. We used the Tet system to induce expression of the wt and mutant forms of PPM1A and specific small interference RNA (shRNA) for its ablation. We hereby demonstrate that overexpression of PPM1A caused cell cycle arrest followed by apoptosis of proliferating PC6-3 cells. Interestingly, in fully differentiated cells PPM1A overexpression did not affect cell growth. We found that the neurite outgrowth process was affected by PPM1A overexpression and its ablation. Furthermore; the PI3K/AKT, ERK and p38 signaling cascades were downregulated in PPM1A overexpressing cells and upregulated in its absence.

## Materials and Methods

### Plasmids

Inducible PPM1A wt or mutant (PPM1A-pcDNA4) expression vectors were previously constructed in our laboratory [11]. As previously published [12], the pSuperRetro-PPM1A was constructed by cloning two annealed oligonucleotides encoding PPM1A specific shRNA into a pSuperRetro vector (kindly provided by R. Agami [25]). The pSuperRetro-LacZ was a gift from Y. Shiloh.

### Cell culture

Trex™PC6-3 (PC6-3) cells were kindly provided by S. Strack. This cell line, used in this study, stably express the Tet repressor from pcDNA6/TR vector [19]. The cells were grown in RPMI 1640 medium supplemented with 8% horse serum (HS), 8% fetal calf serum (FCS), 1% glutamine and 1% pen-strep solution, containing 2 µg/ml blasticidin (Invitrogen). The cell media and serum were purchased from Biological Industries, Israel.

For differentiation, PC6-3 cells were seeded in tissue culture plates coated with mouse tail collagen (kindly provided by R. Stein). Cells were seeded at a relatively low density (1.5×10^5^ cells/60 mm plate) in serum-containing medium. One day after seeding, the medium was replaced with low serum medium (RPMI supplemented with 1% HS and 1% FCS) containing 50 ng/ml NGF. Exposure to vehicle alone (low serum culture medium) was used as the control. The medium supplemented with fresh NGF was replaced every 3 days during the experiments.

### Transfections, infections and establishment of stable transfected cells

PC6-3 cells were transfected with the Lipofectamine™ reagent (Invitrogen) according to manufacturer's instructions. To establish PC6-3 cells stably expressing PPM1A wt or mutant, the cells were transfected with PPM1A wt or mutant-pcDNA4 plasmids. Forty eight hours after transfection cells were seeded for selection into fresh medium containing 200 µg/ml zeocine (Invitrogen). Several stable clones were isolated and analyzed for Tet inducible expression of PPM1A wt and mutant. The suitable clones were grown in selective medium. Tet was obtained from Invitrogen. In all the experiments described below, 250 ng/ml Tet were used to induce PPM1A expression.

In order to establish shRNA stable cell lines, PC6-3 cells were transduced with viruses containing small interfering RNAs (shRNA) against PPM1A (pSuperRetro-PPM1A) and LacZ (pSuperRetro-LacZ). Stable puromycin-resistant cell populations expressing shRNA of PPM1A or LacZ were grown in medium containing 15 µg/ml puromycin (Sigma). Knock down of PPM1A was verified by western blot analysis.

### Colony formation assay

Cells (1000 cells per well) were seeded in 24 wells cell culture clusters. Twenty-four hours later the medium was replaced with Tet containing medium. The medium was changed every 3–4 days until colonies were obtained (10–14 days). The colonies were fixed with 4% formaldehyde in PBS for 20 minutes, washed once with water and stained with Giemsa stain (Sigma Diagnostics diluted 1∶10 and filtered) for 20 minutes.

### Protein Analysis

Cells were lysed in 50 mM Tris, 150 mM NaCl, 5 mM EGTA, pH 7.5, supplemented with Complete™ protease inhibitors (Boehringer-Mannheim), 0.75% NP40, 2 mM Na_3_VO_4_, 50 mM NaF and 10 mM NaPPi. Cell lysis and western blot analysis were carried out as described before [11].

### Antibodies

The following antibodies were used in this study: monoclonal anti-PPM1A antibodies (for production details see [11]), phospho-Ser473-AKT (#44-621G; Biosource), total-AKT (#SAB4500797; Sigma), phospho-Thr180/Tyr182-p38 (#M8177; Sigma), total-p38 (#M0800; Sigma), cleaved-caspase3 (#9661; Cell Signaling Technology), phospho-ERK1(T202/Y204)/ERK2(T185/Y187) (#F1018; R&D Systems), total-ERK (#sc-154; Santa Cruz Biotechnology), p53 (CM5) (#VP-P956; Vector Laborites), MDM2 (4B2 & 2A9; a gift from M. Oren), anti-BrdU (# M 0744; DAKO).

### XTT assay

Cells (1×10^4^ cells/well) were seeded in 96 well cell culture clusters coated with collagen. Next day, the regular medium was replaced with medium containing 1% FCS and 1% HS, with or without NGF (50 ng/ml) and with or without Tet for the indicated time. The cells were then assayed for viability according to manufacturer directions (Biological Industries, Israel).

### Flow Cytometry Analysis and BromoDeoxyUridine (BrdU) staining

Cells were seeded (1×10^6^ cells per 60 mm plates) and Tet was added on the next day. Four hours later 100 µM of BrdU was added for a 6 hours pulse. The cells were harvested at the indicated time periods after Tet addition and fixed with 70% ethanol. The cells were incubated for 20 min with PBS/0.5%Triton X-100 (v/v)/1.5 N HCL at RT, followed by addition of 0.1 M sodium tetraborate (Na_2_BaO_7_.10H_2_O) for 10 minutes. The cells were washed and incubated with 50 µl of anti BrdU antibodies for 1 hour at RT and with 50 µl of anti-mouse IgG FITC antibody for 1 hour at RT. The cells were resuspended in PBS containing 0.1% NaN_3_. For nuclear staining, propidium iodide (50 µg/ml) was added, and the cells were analyzed using fluorescence-activated cell sorter (FACS Caliber, Becton Dickinson).

### Phosphatase activity assays

The activity of PPM1A was measured using the radioactive assay [26]. Briefly, the radioactive phosphatase assay reactions were carried out in a total volume of 30 µl. 15 µg of total extracts were mixed with 5 µM okadaic acid (for the inhibition of other protein phosphatases), 5 mM MgCl_2_, and 10 µl ^32^P-labeled casein (60,000 cpm). After 25 min of incubation at 30°C the reaction was terminated by cold 20% (w/v) TCA and centrifugation. The free ^32^Pi in the supernatant was determined by scintillation counter. Control reactions were carried out in reactions in which protein or magnesium was omitted.

### Statistics

The unpaired 2-tailed Student's t-test was employed in order to compare means for statistical difference. The data in this study is represented as mean ± standard deviation (S.D). Values of P<0.05 were considered significant.

## Results

### Generation and characterization of PC6-3 cell lines stably expressing different levels of PPM1A

**Figure 1:**
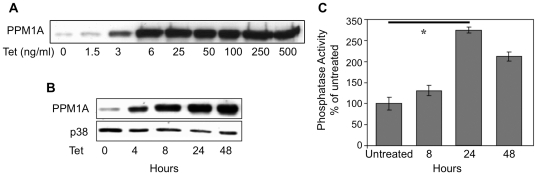
Characterization of stable cell lines expressing PPM1A under the regulation of a Tet-regulated promoter. PC6-3 cells expressing wt PPM1A were incubated for *(* ***A*** *)* 48 hours with increasing concentrations of Tet, or *(* ***B*** *)* for 0, 4, 8, 24, and 48 hours with 250 ng/ml Tet. PPM1A expression was detected using western blot analysis and specific anti-PPM1A antibodies. p38 served as a loading control. The results shown in *(* ***A*** *)* and *(* ***B*** *)* are of representative experiments out of three. *(* ***C*** *)* Extracts from the experiment discussed in *(* ***B*** *)* were assayed for phosphatase activity. The graph shows the average activity of 4 samples from each time point ± standard deviation from one of 3 independent experiments performed. * P-value<0.002.

We have previously shown that PPM1A overexpression inhibits the proliferation of HEK293 cells and induces cell death, thus preventing the establishment of stable clones constitutively overexpressing PPM1A [10], [11]. Similarly, we failed to obtain stable PC6-3 cells overexpressing this phosphatase. Therefore, we constructed Tet inducible cell lines expressing wt and mutant PPM1A. This mutant has lost its catalytic activity due to the replacement of Aspartic acid 239 with Alanine [11]. As shown in Figures 1A and 1B, PPM1A induction depended on Tet concentration and the time after its addition. PPM1A phosphatase activity was significantly enhanced upon its induction (Fig. 1C). The reduction in PPM1A activity 48 hours after Tet induction can be attributed to the death of cells expressing high PPM1A levels. It should be noted that cells overexpressing PPM1A mutant displayed basal phosphatase activity despite the very high level of mutant protein (data not shown).

### PPM1A overexpression inhibits cell proliferation and colony formation

**Figure 2:**
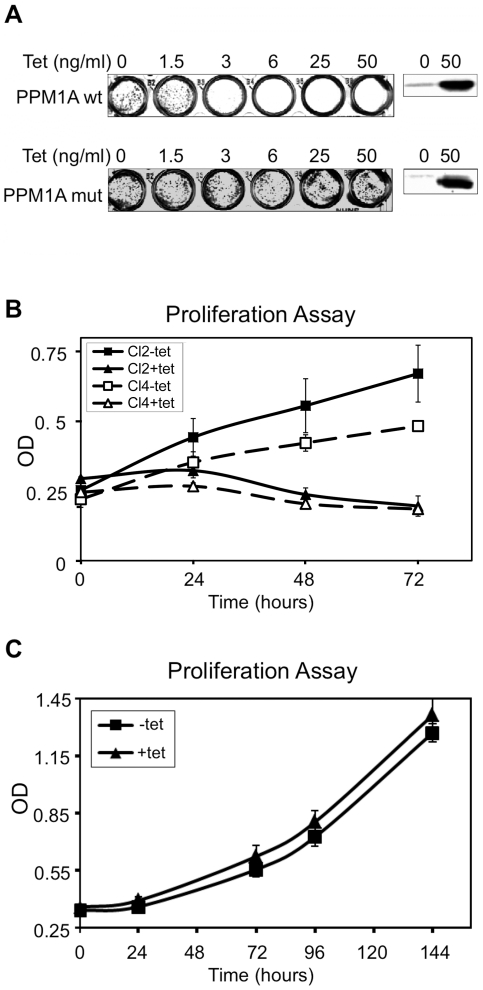
Overexpression of PPM1A wt but not mutant suppresses colony formation and proliferation. *(* ***A*** *)* PC6-3 PPM1A wt or mutant cells were seeded in different Tet concentrations as indicated and grown for 14 days, fixed and stained with Giemsa dye. Cell extracts prepared 48 hours after Tet addition were assayed for PPM1A expression by western blot using anti-PPM1A antibodies. The results shown are of a representative experiment out of three. *(* ***B*** *)* Two clones of PC6-3 PPM1A wt cells (clone 2 and 4) were incubated with or without Tet for 0, 24, 48 and 72 hours. The cells were then assayed for viability using the XTT assay. The average of 6 different wells ± standard deviation from a representative experiment out of three was plotted. *(* ***C*** *)* One clone of PC6-3 cells overexpressing PPM1A mutant was treated as in *(* ***B*** *)*. *cl*- clone.

The effect of PPM1A overexpression on the ability of PC6-3 cells to form colonies was examined. As shown in Figure 2A, PPM1A induction caused a dramatic decrease in colony formation and even at concentration as low as 3 ng/ml Tet there were no viable colonies. Overexpression of PPM1A mutant did not interfere with the ability of the cells to form colonies, demonstrating that PPM1A phosphatase activity is required for its effect on cell survival. Analysis of cell growth using proliferation assay revealed that it was completely inhibited following PPM1A induction (Fig. 2B). However, cells overexpressing mutant PPM1A showed kinetics of cell growth that were similar to the uninduced cells (Fig. 2C).

### PPM1A overexpression induces cell cycle arrest and apoptosis in PC6-3 cell line

**Figure 3:**
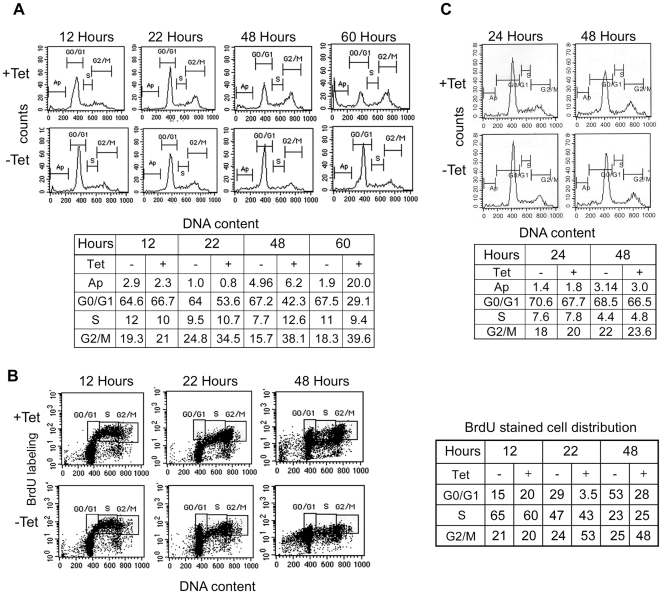
PPM1A overexpression induces cell cycle arrest. FACS analysis *(* ***A*** *)*, and BrdU labeling *(* ***B*** *)* of PC6-3 cells overexpressing PPM1A wt. Cells were treated as described in the “Materials and Methods” and analyzed for DNA content by propidium iodide (PI) staining *(* ***A*** *)* and for BrdU labeling by anti-BrdU antibodies as represented by the dot plot of DNA content (PI) against BrdU incorporation *(* ***B*** *)*. *(* ***C*** *)* FACS analysis of PC6-3 cells overexpressing PPM1A mutant. Cells were incubated with or without Tet, and then analyzed for DNA content by propidium iodide (PI) staining. The numbers represented in the tables indicate % of total cells in *(* ***A*** *)* and *(* ***C*** *)*, and % of total BrdU labeled cells in *(* ***B*** *)*. The experiments in *(* ***A*** *)*, *(* ***B*** *)* and *(* ***C*** *)* were performed at least three times.

We conclude, according to Figure 2, that overexpression of the wt form of PPM1A is toxic to PC6-3 cells. The cell proliferation assay revealed that the total number of cells expressing high levels of PPM1A did not increase (Fig. 2B). However, it was not clear whether those cells were able to proliferate but then died, or whether the cells could not continue with their cell cycle and thus were arrested and then died. To clear this issue we performed BrdU pulse-chase together with FACS analysis of induced and uninduced PC6-3 cells. As shown in Figure 3A, 12 hours after Tet induction, the induced and uninduced cells exhibited similar cell cycle distribution. Differences between their cell cycle patterns are evident as early as twenty two hours after Tet addition. There was a significant increase in the fraction of PPM1A overexpressing cells in the G2/M phase and decrease in their representation in the G0/G1 phase. This phenomenon was more pronounced 48 and 60 hours after Tet addition. Thus, according to the FACS analysis (Fig. 3A), PPM1A overexpression leads to cell cycle arrest at the G2/M phase. The fate of the BrdU pulsed cells verified the G2/M cell cycle arrest. As shown in Figure 3B, 22 hours after Tet induction the BrdU labeled cells expressing basal PPM1A levels transversed from the G2/M to the G0/G1 phase while, the majority of the BrdU labeled PPM1A overexpressing cells were arrested at the G2/M phase (53%) and only a very small fraction (3.5%), of the overexpressing cell moved to G0/G1. Interestingly, 48 hours after Tet induction the fraction of G0/G1 BrdU labeled cells overexpressing PPM1A increased, suggesting that a portion of the arrested cells completed the cell cycle and progressed from G2/M to the G0/G1 phase. Still, the percentage of G0/G1 cells expressing basal PPM1A levels was higher than that of the PPM1A overexpressing cells (Fig. 3B, G0/G1 at 48 hours, 53% uninduced versus 28% Tet induced cells). It should be noted that at later stages a substantial fraction of the PPM1A overexpressing cells underwent apoptosis (Fig. 3A 60 hours) and died (Fig. 2). We next examined whether PPM1A phosphatase activity is important to the cell cycle arrest phenomenon. As shown in Figure 3C overexpression of the mutant form of PPM1A for 24 and 48 hours had no effect on the cell cycle distribution. Altogether, we conclude that the inhibition of the proliferation of PPM1A overexpressing cells which was shown in Figure 2B is mainly due to cell cycle arrest at the G2/M phase, and cells unable to proceed with their cell cycle undergo apoptosis, as was previously shown in HEK293 cells [11]. It is not clear whether those cells that proceeded to the G0/G1 underwent apoptosis or continued to the G2/M phase and then apoptosed. However, eventually, all PPM1A overexpressing cells died (Fig. 2A and 2B).

**Figure 4:**
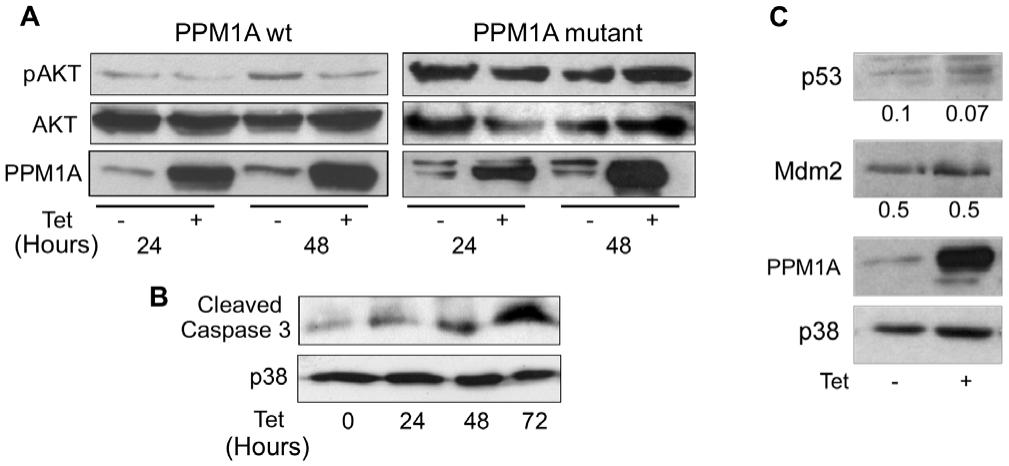
PPM1A overexpression affects cell cycle and survival markers. *(* ***A*** *)* PC6-3 cells overexpressing wt or mutant PPM1A were incubated with or without Tet for the indicated times. The cells were then harvested and lysed. Cell extracts were analyzed by western blot for levels of phosphorylated AKT, total AKT and PPM1A. Note that PPM1A mutant migrates faster than the wt on the gel ([10]) and that even in the absence of Tet there was a slight expression due to leakiness of PPM1A mutant. PPM1A wt cells were analyzed in the same manner for levels of *(* ***B*** *)* cleaved caspase 3 and p38. *(* ***C*** *)* PC6-3 cells overexpressing PPM1A were incubated with or without Tet for 24 hours. The cells were then harvested and lysed and cell extracts were analyzed for levels of p53, Mdm2, PPM1A and p38 by western blot. The numbers represent the ratio of p53/p38 (0.1, 0.07) and Mdm2/p38 levels (0.5, 0.5). The blots presented in *(* ***A*** *) (* ***B*** *)* and *(* ***C*** *)* are from representative experiments out of three performed.

Several studies have demonstrated the requirement for AKT activity in the G2/M phase transition [27], [28]. Indeed, forty eight hours after PPM1A induction there was a decrease in the level of AKT phosphorylation on serine 473 which is required for the activity of AKT as a survival factor (Fig. 4A). Overexpression of the mutant form did not affect AKT phosphorylation levels (Fig. 4A). Cleaved caspase 3 serves as an apoptotic marker [29] and indeed its elevated levels in PPM1A overexpressing cells indicate the enhanced apoptosis (Fig. 4B). Altogether, our cell cycle and biochemical data demonstrate that in replicating PC6-3 cells PPM1A overexpression arrests cell cycle progression and induces apoptosis.

In HEK293 the growth arrest and apoptosis induced by PPM1A overexpression were partially mediated by its effect on p53 protein [11]. This was shown by p53 elevated protein levels upon PPM1A induction and increase in its activity as a transcription factor. However, in PC6-3 cells the elevated PPM1A levels did not increase the level of p53 or its transcriptional target Mdm2 (Fig. 4C).

### PPM1A overexpression inhibits PC6-3 cell proliferation during differentiation, but does not induce apoptosis of fully differentiated cells

**Figure 5:**
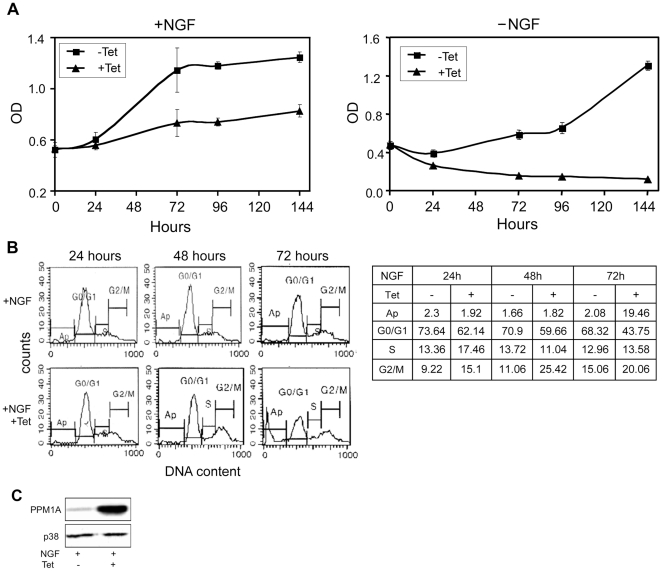
PPM1A overexpression and PC6-3 cells differentiation. *(* ***A*** *)* Cell proliferation assay. PC6-3 PPM1A wt cells were incubated with or without NGF and with or without Tet. The cells were then assayed for viability at the indicated times using XTT assay. The average of 6 different wells ± S.D. from a representative experiment was plotted. *(* ***B*** *)* FACS analysis of PC6-3 cells preincubated with Tet for 24 hours followed by addition of NGF for the indicated times. The table represents % of total counted cells of one experiments out of three performed. *(* ***C*** *)* Western blot analysis of extracts from cells treated for 144 hours with NGF with or without Tet, and assayed for PPM1A expression level with anti-PPM1A antibodies.

**Figure 6:**
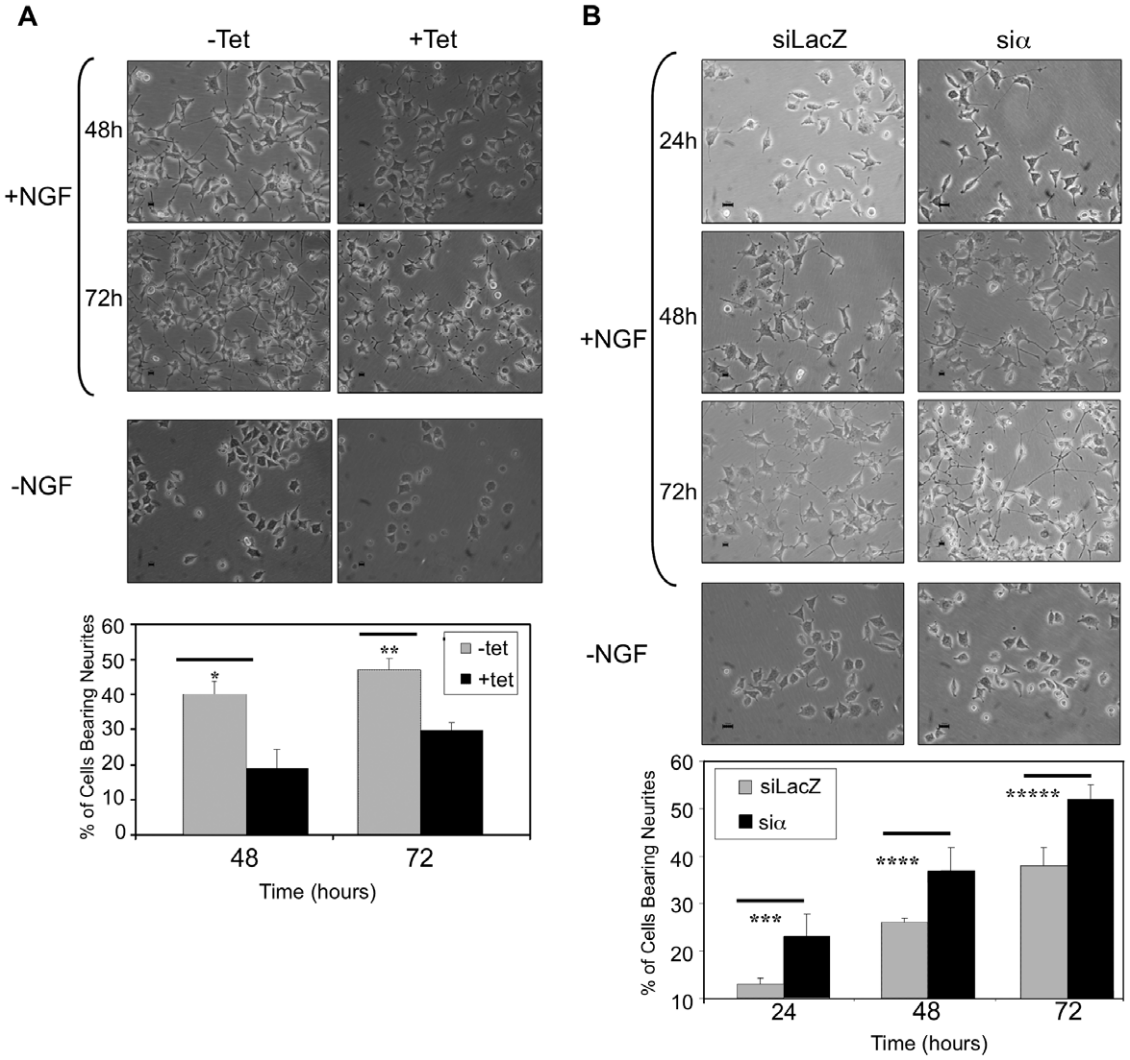
PPM1A expression affects neurite outgrowth. *(* ***A*** *)* PC6-3 PPM1A wt cells were seeded on collagen coated plates and incubated with or without NGF and Tet. *(* ***B*** *)* PC6-3 siLacZ and siα cells were seeded in the same manner and treated with or without NGF. Phase contrast images were taken at the indicated time periods. The histogram represents quantification of neurite formation from one out of three independent experiments. Cells with processes twice longer than cell body were counted as positive. At least 100 cells were counted from 5 different areas of each plate and the average numbers are represented with standard deviation. P-Values: * P-Values<0.01; ** P-Values<0.0005; *** P-Values<0.03; **** P-Values<0.03; ***** P-Values<0.05.

Upon NGF addition, PC6-3 cells differentiate into neuronal like cells. During this process the cells proliferate rapidly for approximately 3 days, and then, concomitantly with the appearance of neuronal-like extensions, the cells arrest at the G0/G1 phase. To study the role of PPM1A in the differentiation process, cells expressing inducible PPM1A were incubated with or without NGF in the presence or absence of Tet. Cells expressing basal PPM1A levels underwent rapid replication for 72 hours followed by proliferation arrest and differentiation into neuronal like cells (Fig. 5A and Fig. 6). PPM1A overexpression affected the cells during their proliferation phase and led to the cessation of cell division (Fig. 5A). Indeed, cell cycle analysis of the NGF-treated PPM1A overexpressing cells revealed that first, the PPM1A overexpressing cells accumulated in the G2/M phase and later on, 72 hours after Tet addition, a fraction of the cells underwent apoptosis (Fig. 5B). Interestingly, afterwards, the fully differentiated cells treated 6 days with NGF and Tet were not further affected by PPM1A overexpression (Fig. 5A, left), even though it was still highly expressed (Fig. 5C) and exhibited higher phosphatase activity than that of untreated cells or uninduced NGF-treated cells (data not shown). We have also examined the effect of PPM1A induction on differentiating cells. To this end, the cells were pretreated with NGF and on the third or fifth days of the NGF treatment PPM1A was induced by Tet addition. Our findings demonstrated that PPM1A overexpression had no effect on the viability of the cells (data not shown).

### PPM1A overexpression delays neurite extension during the differentiation process

Next we asked whether PPM1A affects neurite extension and cellular morphology during the differentiation process. To this end, cells were seeded on collagen coated plates, and NGF was added 24 hours later with or without Tet. As seen in Figure 6A, 48 and 72 hours following NGF addition PPM1A overexpressing cells exhibited shorter extensions than uninduced cells expressing basal PPM1A levels. However, 7 days after NGF addition both cell types displayed similar extension length (data not shown).

The interplay between the toxic effect of PPM1A overexpression and the protective effect of NGF should be noted. On one hand there is a dramatic reduction in the number of undifferentiated PPM1A overexpressing cells (Fig. 6A −NGF +Tet) in comparison to cells exhibiting basal PPM1A levels (Fig. 6A −NGF −Tet). On the other hand, NGF treatment reduced the toxic effect of PPM1A overexpression (Fig. 6A +NGF +Tet 48 and 72 hrs). Still, the number of differentiated cells was lower than in the NGF treated cells expressing basal PPM1A levels (Fig. 6A +NGF −Tet 48 and 72 hours).

### PPM1A knock down cells continue to proliferate during PC6-3 differentiation

**Figure 7:**
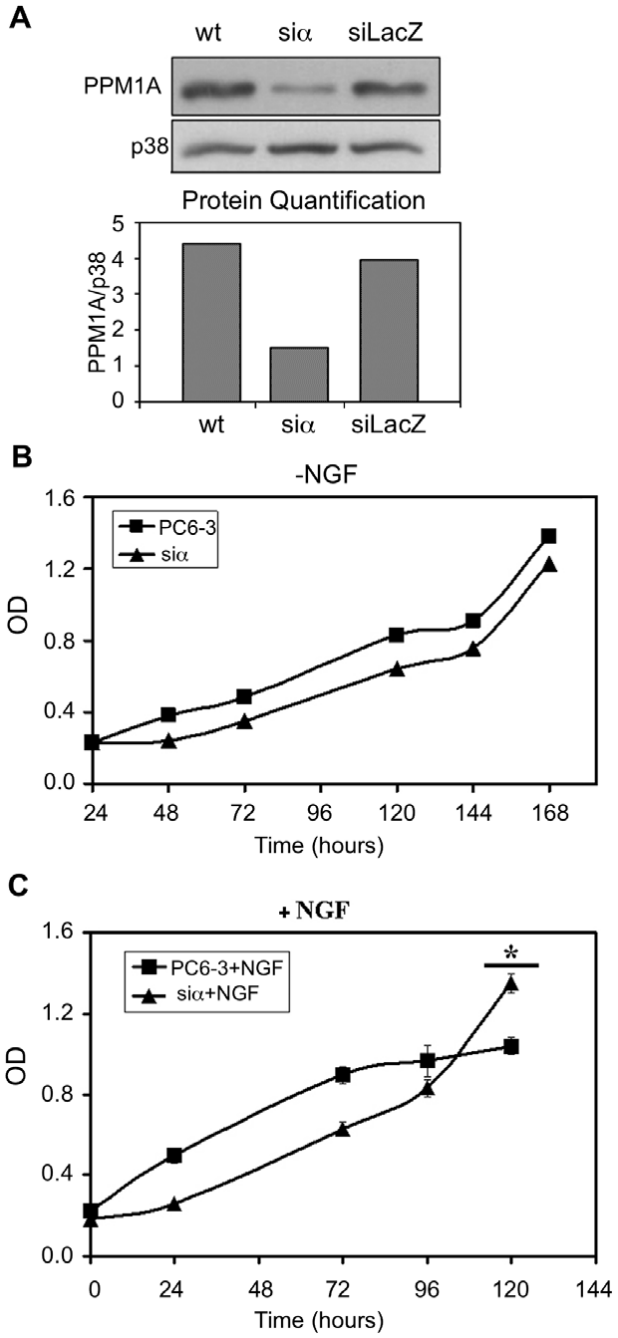
Proliferation assay of PPM1A knock-down cells. *(* ***A*** *)* PC6-3 cells were transduced with pSuperRetro-PPM1A or pSuperRetro-LacZ. Forty eight hours later cells were selected by the addition of puromycin. The protein levels of PPM1A were analyzed by western blot. The graph represents the quantification of PPM1A levels. *(* ***B*** *)* and *(* ***C*** *)* Proliferation assay of PPM1A knock-down cells compared to normal cells in the absence *(* ***B*** *)* or presence *(* ***C*** *)* of NGF. The average of 6 different wells ± standard deviation from a representative experiment out of 3 independent experiments that were performed was plotted. * P-value of 120 hours <0.002.

To investigate the role of endogenous PPM1A in the differentiation process we established PPM1A knockdown cells with markedly reduced endogenous PPM1A levels (siα cells), and siLacZ cells that served as control (Fig. 7A). Note that though the expression of the endogenous PPM1A was marginal in the siα cells, we could not monitor the decrease of its activity in the knockdown cells since this assay detects the phosphatase activity of all PPM1 paralogs and is not specific to PPM1A protein. We examined the proliferation pattern of PPM1A knockdown cells and found that in the absence of NGF the cells exhibited a proliferation pattern similar to that of normal cells (Fig. 7B). However upon NGF addition there were differences between the two cell types. Seventy two hours after NGF addition the wt cells underwent growth arrest while the siα cells continued to proliferate (Fig. 7C). Furthermore, in the PPM1A ablated cells neurites appearance was accelerated (Fig. 6B). Interestingly, seven days after NGF addition both cell types displayed similar morphology.

### PPM1A is involved in NGF signaling pathways during differentiation

**Figure 8:**
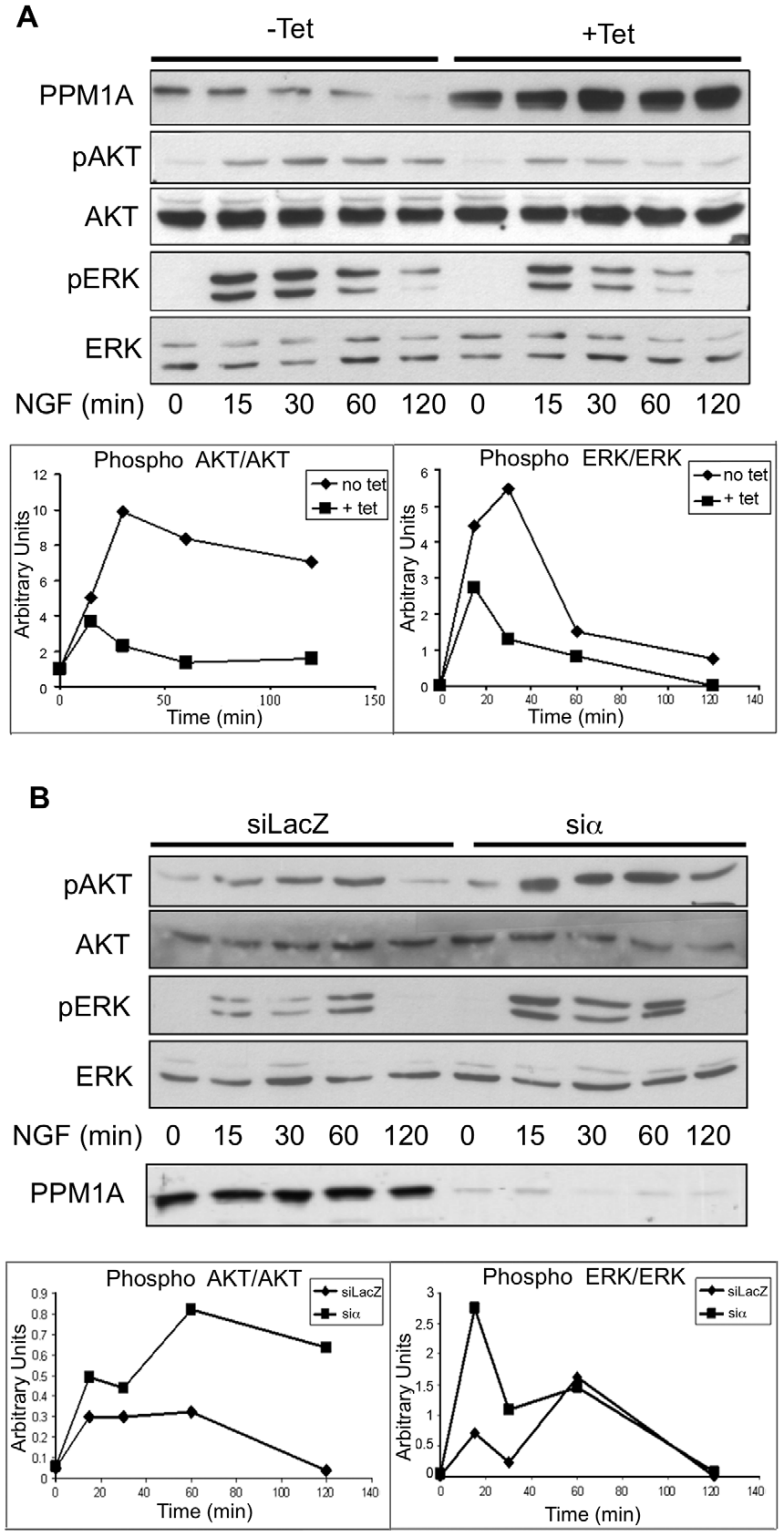
PPM1A over and underexpression and cell signaling during PC6-3 cells differentiation. *(* ***A*** *)* PPM1A wt cells were incubated with or without Tet for 24 hours and then NGF was added for the indicated times. *(* ***B*** *)* PC6-3 PPM1A knock-down cells and control cells were incubated with NGF for the indicated times. The cells were harvested and lysed. Cell extracts were analyzed by western blot using the indicated antibodies. Please note that the AKT and PPM1A panels in *(* ***B*** *)* are from different gels of the same experiments. The graphs represent quantification of the blots shown in *(* ***A*** *)* and *(* ***B*** *)*. The results shown are from representative experiments out of 3 independent that were performed.

It is well established that AKT, ERK and p38 activation is part of the differentiation process of PC12 cells. We examined the phosphorylation of AKT and ERK in NGF treated cells overexpressing PPM1A and in knockdown cells. PPM1A overexpression led to decreased levels of phosphorylated AKT and ERK, while the opposite picture emerged upon the analysis of PPM1A ablated cells (Fig. 8A and 8B).

**Figure 9:**
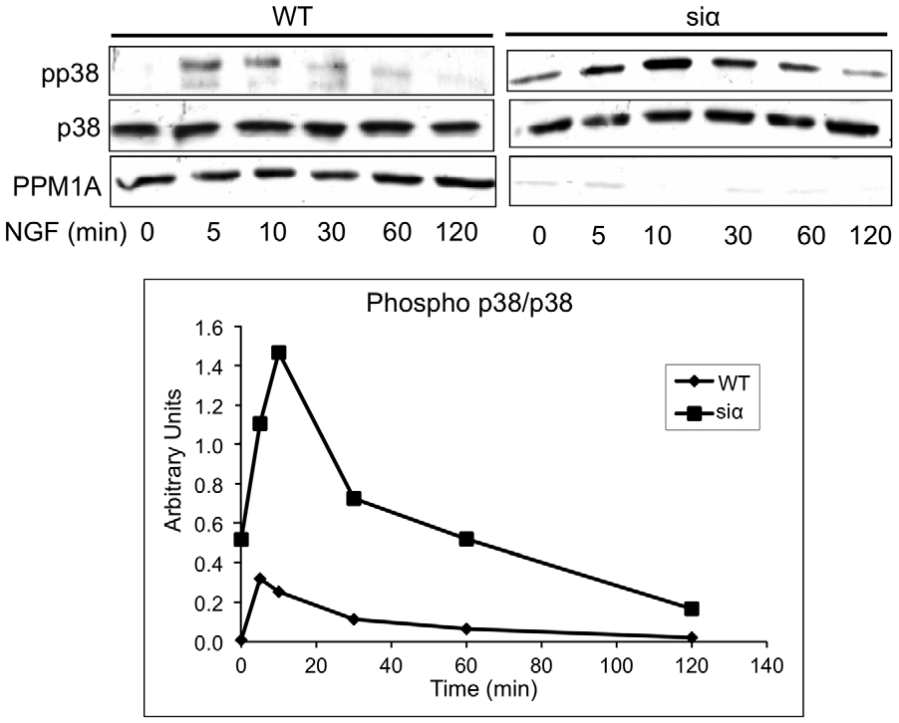
Phosphorylation of p38 during NGF treatment is increased in PPM1A underexpressed PC6-3 cells. PC6-3 PPM1A knock-down cells and control cells were incubated with NGF for the indicated times. The cells were harvested and lysed. Cell extracts were analyzed by western blot using the indicated antibodies. The graph represents quantification of the phospho-p38 levels. The results shown are from representative experiments out of 3 independent that were performed.

p38 is an established substrate of PPM1A, and the interaction between these two proteins was previously demonstrated [5]. The role of PPM1A in the p38 pathway during NGF exposure was examined. In knockdown cells, the levels of phospho-p38 were dramatically higher and persisted for a longer period in comparison to the wt cells (Fig. 9). Accordingly, PPM1A overexpression resulted in reduced phospho-p38 levels in NGF treated cells (data not shown).

Altogether, these findings demonstrate that PPM1A is involved in the AKT, ERK and p38 signaling, and its activity as phosphatase controls the phosphorylation state of these important proteins which participate in the NGF signaling pathways.

## Discussion

PC12 cells are used as a model system for the study of neuronal differentiation, survival and signaling. In this report we propose a new role for PPM1A in these pathways using PC6-3 cells, a subclone of PC12 cell line.

### PPM1A, cell cycle arrest and apoptosis of PC12 cells

Overexpression of different members of the PPM1 family in various cell lines was shown to cause cell cycle arrest and apoptosis [9],[11],[30],[31]. In this study we have examined the effect of PPM1A on differentiation, viability and survival of PC6-3 cells. We have shown that PPM1A overexpression leads to immediate cell cycle arrest at the G2/M phase. This arrest was evident by the increased fraction of cells in G2/M phase detected by direct cell cycle analysis and the BrdU pulse chase data. Later on a fraction of the cells managed to exit G2/M and began a new cell cycle. It is not clear whether the cells were arrested at the G0/G1 and then underwent apoptosis, or continued to the G2/M and then apoptose. The cell cycle arrest corresponds to the cessation of proliferation that was observed in PPM1A overexpression cells. Eventually, the halted cells undergo apoptosis. We suggest that the primary effect of PPM1A is the cell cycle arrest; while the apoptotic response, shown by the cell cycle analysis and enhanced caspase 3 cleavage, is due to a secondary effect resulting from the inability of the arrested cells to resume advance in the cell cycle. In long term, PPM1A induction leads to reduction in cell number and colonies formation. We conclude that these effects are mediated by PPM1A activity as phosphatase, since the mutant form of the protein, which lacks phosphatase activity, failed to affect the cell cycle distribution and cell viability.

The low level of phosphorylated AKT upon PPM1A induction also supported the G2/M phase arrest caused by PPM1A overexpression, as the involvement of AKT in the G2/M transition and survival pathways was previously documented [27],[28]. In addition, Lee et al. demonstrated that in PC12 activated-AKT was necessary to the cells proliferation and cell cycle progression [32]. It is known that upon its phosphorylation and activation by the PI3K, AKT activates various anti apoptotic second messengers and deactivates pro-apoptotic second messengers, such as BAD and the forkhead transcription factors family [33],[34]. Furthermore, AKT inactivation was demonstrated during cell death and its down-regulation enhanced basal cell death [35]. We propose that the effect of PPM1A on the cell cycle and cell death might be partly attributed to its effect on AKT dephosphorylation and the activation of forkhead and BAD.

Inhibition of the PI3K/AKT survival pathway also occurs in PC12 cells, following various stress responses such as oxidative and ER stress, and serum deprivation. Some of these modes of stress can lead to neurodegenerative diseases, which are involved in neuronal death. By affecting the PI3K/AKT pathway PPM1A might be engaged in regulating these cellular responses and thus may play a role in neurodegenerative diseases.

### PPM1A, PC12 cells differentiation and signaling

The involvement of tyrosine phosphatases in the differentiation process into neuronal like cells is highly investigated. However, the role of serine/threonine phosphatases, especially the PPM1 family, in this process is less studied. The high expression of PPM1A in the brain, the role of PPM1A and PPM1B in neuroprotection and neurodegeneration [36],[37], the involvement of PPM1 family members in other differentiation processes [38],[39], coupled with our and previous findings on the effect of PPM1A in cell cycle progression and the regulation of the PI3K/AKT pathway [39], prompted us to examine whether PPM1A is engaged in the differentiation of PC12 cells. To this end we used two complementary approaches and studied PPM1A overexpression and its ablation in the NGF treated cells. While its overexpression was toxic to proliferating cells but did not affect the fully differentiated cells, PPM1A knockdown resulted in delayed growth arrest and extended proliferation phase upon exposure to NGF. In view of our finding that PPM1A overexpression leads to G2/M arrest and still facilitates the transit of a fraction of the cells to G0/G1 (Fig. 3), we suggest that upon NGF treatment of Tet induced cells there is a competition during the proliferating phase of the NGF treatment between the signals induced by PPM1A overexpression and NGF on the cells fate. Cells arrested in the G2/M due to PPM1A overexpression will probably undergo apoptosis; however, cells that escape and proceed to the G0/G1 might be protected from the PPM1A toxic effect and will differentiate. This competition will last as long as the cells are in the proliferating stage. Then, as the cells stop proliferating and start to differentiate they can be protected against PPM1A toxicity and they will probably be found in a cell cycle phase protected from PPM1A toxic effect such as G0 phase. On the other hand, basal PPM1A levels are necessary for the normal proliferating pattern of the differentiating cells, since its ablation hampered the proliferating halt of the differentiating cells (Fig. 7).

We have demonstrated here that in NGF treated cells the levels of PPM1A determined the phosphorylation state of AKT, ERK and p38, proteins that participate in the differentiation process. While PPM1A overexpression led to reduced phosphorylation, its ablation resulted in elevation of their phosphorylation. The signaling pathway(s) through which PPM1A affects the phosphorylation of these proteins are still unknown. One possibility is that this phosphatase is directly involved in the dephosphorylation of those proteins. This hypothesis is in accordance with previous studies in which p38 was shown to be dephosphorylated and regulated by PPM1A [5]. However, one should consider the possibility that PPM1A might regulate an upstream protein(s) which control the signaling pathways of those three proteins. As mentioned in the introduction, AKT, ERK and p38 are all signaling cascades controlled by the activity of the receptor tyrosine kinase TrkA. TrkA is a member of the Trk gene family which is composed of three related receptors, TrkA, TrkB and TrkC. TrkA binds specifically to its ligand NGF, and this binding induces receptor dimerization, auto phosphorylation on specific tyrosine residues within its intracellular domain and the receptor activation [40],[41]. Since PPM1A altered these TrkA signaling pathways, it is possible that it is engaged directly or indirectly in TrkA activation and thus affects its downstream signaling cascades. In earlier studies using COS7 and HEK293 cells lacking Trk receptor, PPM1A did not affect ERK or AKT phosphorylation ([42], and P. Ofek unpublished results). These findings support the hypothesis that PPM1A acts through TrkA.

There is evidence supporting the hypothesis that altered AKT phosphorylation under NGF exposure conditions results in inability of PC12 cells to generate proper neuronal extensions and differentiate normally into neuronal like cells [43]. Overexpression of the tumor suppressor PTEN inhibited the development of neuronal phenotype in PC12 cells [44], while depletion of PTEN did not prevent neuronal differentiation [45]. In addition, overexpression of the PP2A regulatory subunit Bγ promoted neuronal differentiation of PC6-3 cells without NGF addition [19]. Similarly, we observed that neuronal extension was also affected by PPM1A levels. PPM1A ablation led to earlier neuronal outgrowth while its overexpression delayed their appearance.

NGF treatment of PC12 results in short term and long term effects on the cells: in the short term NGF stimulates different signaling cascades immediately upon its addition and in the long term it leads to growth arrest, morphology changes and differentiation. Our findings demonstrate that PPM1A participates in both the immediate and long term NGF effects. It is possible that the involvement of PPM1A in the differentiation process (its effect on the neuronal extensions and cell proliferation) is a consequence of its immediate effects on the NGF stimulated cellular signaling.

On the whole, our results indicate that PPM1A has an important role in the survival and differentiation of PC12 cells into neuron-like cells. In concert with earlier studies on the involvement of PPM1A and PPM1B in neurodegeneration and neuroprotection [36], [37], [46] PPM1A is probably required during embryonic development of the central neuronal system (CNS) and may participate in neuronal death in neurodegenerative diseases.
